# Research on inhibitory effect of mixed suppressants CaCO_3_, KCl, and K_2_CO_3_ on coal dust explosion pressure

**DOI:** 10.1038/s41598-024-58017-7

**Published:** 2024-03-27

**Authors:** Tianqi Liu, Kenan Liu

**Affiliations:** School of Safety Engineering, Shenyang Aerospace University, Shenyang, 110136 Liaoning China

**Keywords:** Coal dust, Inhibition effect, Maximum pressure, Explosion suppressant, Chemical safety, Energy, Chemical engineering

## Abstract

To discuss the inhibitory effect of micrometer scale coal dust explosion pressure, three types of explosion suppressants are selected for mixed explosion suppression. The results indicate that the coal dust explosion process includes three stages: accelerated and decelerated energy release, as well as energy dissipation. When using explosive suppressants, K_2_CO_3_ has the greatest inhibitory effect on coal dust explosion, followed by KCl, and CaCO_3_ has the smallest effect. The K_2_O, K_2_O_2_, and KOH generated by the thermal decomposition of K_2_CO_3_ can also block the heat transfer of coal dust, playing a good role in suppressing explosions. The explosion suppression effect of mixing CaCO_3_ and K_2_CO_3_ is better than that of mixing CaCO_3_ and KCl, and is worse than the explosion suppression effect of using K_2_CO_3_ alone. The synergistic effect of KCl and K_2_CO_3_ mixed explosion suppression makes the suppression effect better than using K_2_CO_3_ alone. This is because KCl generates K_2_O during pyrolysis, promoting the dynamic equilibrium of K_2_CO_3_ explosion suppression process. This makes mixed explosion suppression more worthy of attention and adoption when considering purchase costs.

## Introduction

In today's energy security field, coal dust explosion accidents still seriously plague safety production. In the process of coal mining and processing, some unintentional human negligence and errors can cause significant accidents. The frequent occurrence of coal mine explosions is one of the major coal mine disasters. Industrial dust particles are generated during coal transportation, which may seem small but contain enormous energy^1,2^. There are three main reasons for the enormous energy of coal dust explosions. The first reason is that the particle size of coal dust is very small, usually at the micrometer or even nanometer scale, which is difficult to observe with the naked eye. It can not only cause explosion accidents, but also lead to miners suffering from pneumoconiosis. The second reason is that the number of coal dust particles is usually very large. In confined spaces, the energy contained in a large number of coal dust particles will continue to accumulate, which is very dangerous^3–5^. The third reason is that these coal dust particles are very small, so they are easily suspended, and suspended coal dust clouds are one of the necessary conditions for coal dust explosions^6–8^. Therefore, the study of coal dust explosion suppression is very important, and effective explosion suppression methods can reduce the power of explosions and reduce casualties.

In the field of dust explosion dynamics, explosion characteristics and explosion suppression characteristics are two hot topics. Industrial explosions mainly include gas explosions and dust explosions. In coal mines, gas explosion refers to methane explosion. Methane explosion is a chemical reaction that emits light and heat, and is a typical combustion process. Coal dust explosion not only involves the combustion of combustible gases, but also the combustion of combustible particles, making its explosion mechanism more complex. Currently, related research is still being extensively conducted^9–19^. Coal dust explosion belongs to the combustion process of multiphase flow, and the duration of the explosion is very short, making the explosion process difficult to capture. Scholars can obtain the intensity characteristics of gas and coal dust explosions through continuous experiments and have achieved certain results^20–26^. Numerical simulation technology has also been developed to explore the characteristics and propagation process of coal dust explosions. The continuous optimization and improvement of relevant simulation models have provided great help in improving simulation accuracy and saving simulation time^27,28^. These research methods can also be applied to the study of coal dust explosion suppression.

The inhibitory effect of different explosion suppressants on coal dust explosions varies. During the formation of coal in the crust, the degree of metamorphism of coal varies due to different formation times, resulting in different suppression characteristics of coal dust explosions. At the same time, there are also many types of explosion suppressants, most of which are the main components used in industrial fire extinguishing agents and can effectively suppress coal dust explosions^29–31^. By mixing coal dust with explosion suppressants, scholars have preliminarily obtained some inhibitory effects of explosion suppressants on coal dust explosions, including how to completely suppress explosions and prevent them from happening again. These studies are of great significance for understanding the characteristics of coal dust explosion suppression^32–36^. With continuous research, some new types of explosion suppressants have also been developed. Usually, their suppression effect is very good and they can effectively control the occurrence of explosions. However, the disadvantage is that the cost is too high. Many coal mining enterprises do not use high costs for explosion prevention, so they have certain limitations in application ^37,38^. Based on the above analysis, researching effective and economical explosion suppression methods is still an important task, which is also the starting point of this paper.

Therefore, in this article, the authors mainly consider the hazards of coal dust explosions and how to effectively suppress them, and conduct relevant research through experimental means. In the preliminary research of the authors, theoretical and experimental studies have been conducted on the characteristics of explosion ignition, flame propagation process, and the influence of related factors^39–43^. The author also obtained some inhibitory effects of explosion suppressants on the intensity of coal dust explosions, but these results are limited to the use of single component explosion suppressants^44–46^. Furthermore, the authors believe that further research is needed in the field of coal dust explosion and its suppression. The research results on the suppression effect of coal dust explosion under different scheme conditions are not very comprehensive. Therefore, starting from the premise of mixed explosion suppressants, the authors discuss the characteristics of coal dust explosion suppression under different mixed explosion suppressant conditions in this article. The research results are of great significance for understanding the characteristics of coal dust explosion under different explosion suppression scheme conditions and also provide guidance for the prevention of industrial coal dust explosion disasters.

## Experimental scheme

### Dust explosion device

**Figure 1:**
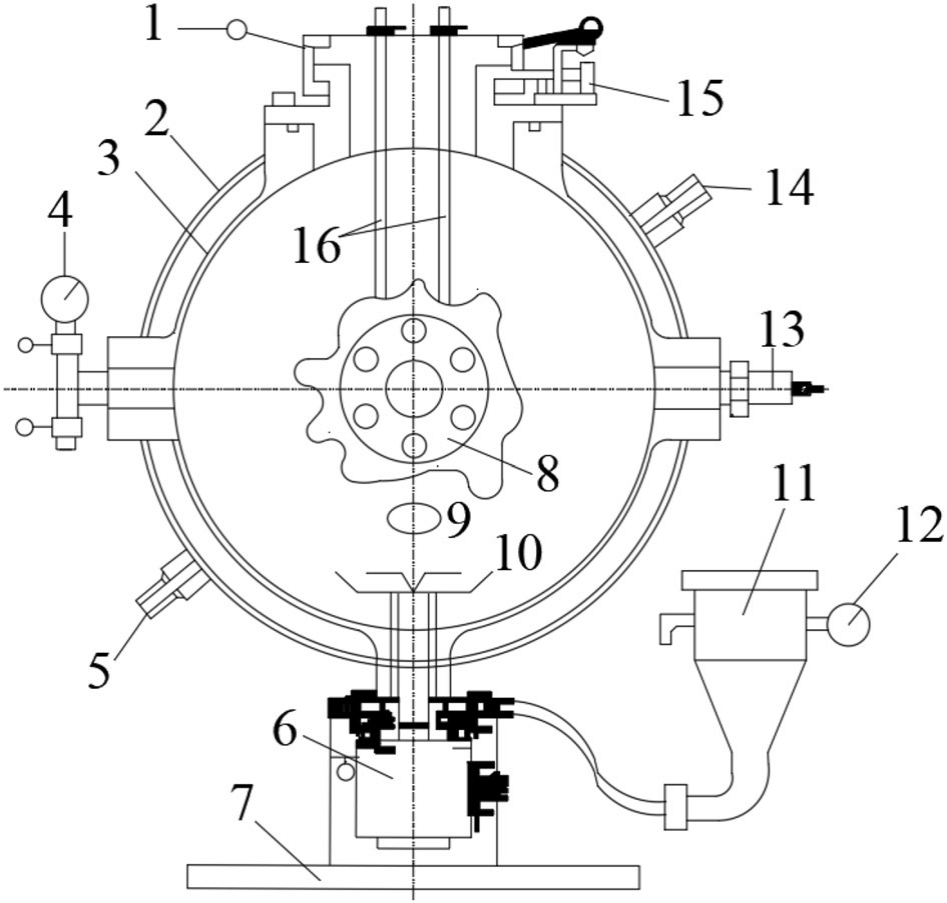
Structure of experimental device. 1 sealing cap; 2 outer side of mezzanine; 3 inside of mezzanine; 4 vacuum gauge; 5 outlet of circulating water; 6 mechanical two-way valve; 7 base; 8 observation window; 9 vacuum hole; 10 dispersion valve; 11 dust storage tank; 12 pressure gauge; 13 pressure sensor; 14 inlet of circulating water; 15 safety limit switch; 16 ignition rod.

In the experiment of this article, the dust explosion experimental device used is the explosion chamber of the sphere. The structure of the experimental device is shown in Fig. 1. It mainly consists of sixteen parts, and the internal space of its explosion chamber is twenty liters. This device was first invented by German scholars and later improved by American scholars, ultimately forming its current form. It is also one of the commonly used industrial gas and dust explosion experimental devices internationally. Scholars from different countries have used this experimental device, and only through experimental testing can the dust explosion data results be comparable. Meanwhile, the devices used in different countries comply with relevant international standards. The advantage of this device is that it is easy to operate and can be remotely controlled.

The main parameters during the experiment are as follows: the energy of the two ignition heads is 10 kJ, the ignition delay time is 0.1 s, and the pressure for spraying dust is 2 MPa. These parameters are the basic data that can ensure the occurrence of coal dust explosions under conventional experimental conditions. The ignition energy is 10 kJ, mainly because this energy can successfully ignite coal dust. The ignition energy of coal dust is much greater than that of gas. If the energy is too small, coal dust will not explode. Experimental personnel can also make corresponding adjustments according to specific experimental schemes, as long as the safety of explosion conditions and the reliability of experimental results are guaranteed.

### Experimental coal dust

**Figure 2:**
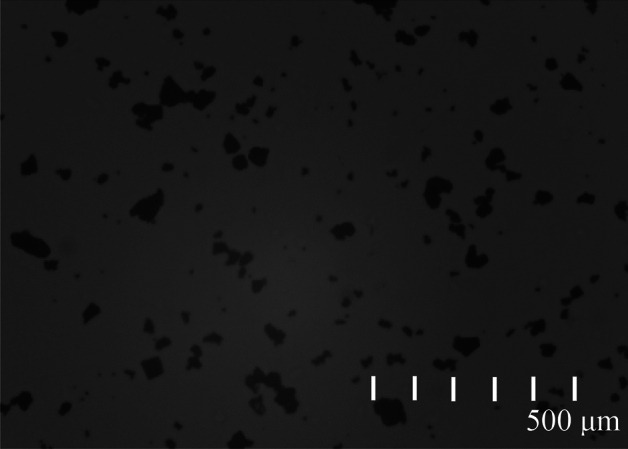
Distribution of coal dust particles.

The particles of coal dust samples are on the micrometer scale. In general, the explosiveness of coal dust samples at the micrometer scale is the most obvious. Some scholars judge the explosiveness of coal dust based on the particle size, and believe that coal dust particles with a diameter of 75 μm have the highest explosiveness. If the particle size of coal dust is too large, it is difficult to cause coal dust explosion. In order to clearly display the morphology and size of coal dust particles, coal dust particle size analysis experiments were conducted, and the results obtained are shown in Fig. 2. It shows the distribution of observed coal dust particles.

In order to obtain the main components of the coal dust sample, experimental tests were conducted, and the results are shown in Table 1. The composition of coal dust samples includes four parts, namely moisture, ash, volatile, and fixed carbon. Their sum is 100%. Moisture refers to the percentage of water released from coal dust under heating conditions compared to the original coal sample mass. The moisture content obtained from the experiment is 5.15%, which gives the coal dust sample a certain viscosity. Ash content refers to the percentage of the mass of substances that cannot participate in chemical reactions during coal dust combustion or explosion compared to the original coal dust mass. The ash content of the coal sample can be obtained to be 16.78%, and these components actually play a role in inhibiting chemical reactions during coal dust combustion or explosion. Among the above two components, both moisture and ash essentially have a suppressive effect on the combustion and explosion reactions of coal dust samples.

**Table 1:** Main components of coal dust samples used in the experiment.

Coal sample composition	Components that suppress explosions		Components that contribute to explosions	
	Moisture (%)	Ash (%)	Volatile (%)	Fixed carbon (%)
Value of content	5.15	16.78	31.36	46.71

### Experimental explosion suppressants

**Figure 3:**
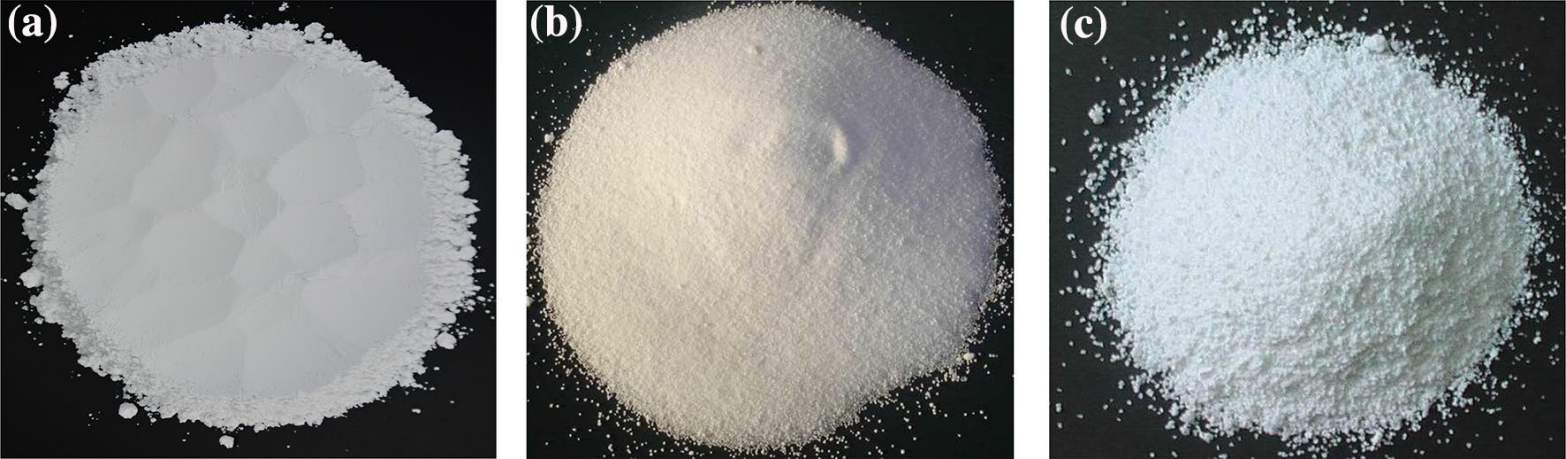
Three types of inert explosion suppression dust selected in the experiment.

In this study, three types of explosion suppression dust were used, namely CaCO_3_, KCl, and K_2_CO_3_. The images of three types of explosion suppression dust samples after preparation are shown in Fig. 3. It can be seen that they are all white solid crystals at room temperature and are easily made into white powders. Among them, CaCO_3_ is relatively the cheapest in price. KCl and K_2_CO_3_ are also common chemical agents in industry and can be easily purchased. They are also convenient for transportation and storage, which is the main reason for choosing these three inert dusts as explosion suppression dust for experimental research. The particle size of the three types of explosion suppression dust is also in the micrometer range.

## Results and discussion

### Explosion pressure of coal dust with micrometer particle size

**Figure 4:**
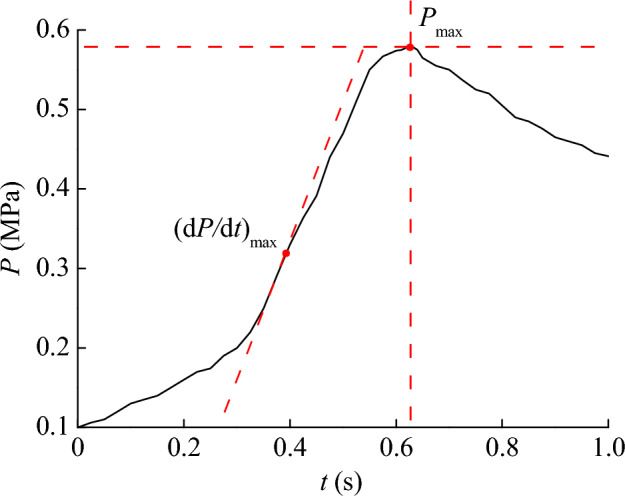
Explosion pressure curve of coal dust with micrometer particle size.

By using the explosion pressure experimental device to test the explosion characteristics of coal dust, the pressure curve after the explosion can be obtained. It should be noted that the mass of coal dust used in each explosion experiment is calculated based on the volume of the explosion space and the concentration of coal dust cloud mass. The mass concentration of coal dust cloud is equal to the mass of coal dust divided by the volume of the explosion space. In this part of the experiment, the mass of coal dust in the explosion experiment is 10 g. The volume of the explosion space is 20 L, which is 0.02 m^3^, so the mass concentration of coal dust clouds in the explosion space is 500 g/m^3^. This mass concentration is also a key factor in meeting the conditions for coal dust cloud explosion. So the obtained coal dust explosion pressure curve is shown in Fig. 4. It displays the process of pressure changes over time after an explosion, which can be used to analyze the maximum pressure and the rate of maximum pressure rise. Among them, the maximum pressure is abbreviated as *P*_max_, and the maximum rate of pressure rise is abbreviated as (d*P*/d*t*)_max_. In Fig. 4, *t* represents the time after the explosion, and *P* represents the explosion pressure.

In order to obtain specific explosion pressure data, the data on the pressure curve was extracted, and the results are shown in Table 2. At 0.375 s, the maximum pressure rise rate of coal dust explosion increased to its maximum. Subsequently, at 0.625 s, the maximum pressure of the coal dust explosion increased to its maximum. The moment corresponding to the maximum pressure rise rate occurs before the moment corresponding to the maximum pressure. The interval between two moments is 0.25 s. During this 0.25 s period, although the rate of increase in coal dust explosion pressure was decreasing, the explosion pressure continued to increase, indicating that the energy of the explosion was still being rapidly released. The test shows that at 0.375 s, the maximum pressure increase rate of the explosion is 32.90 MPa/s, at 0.625 s, the maximum explosion pressure is 0.58 MPa. The above explosion pressure test results provide an important basis for the study of coal dust explosion suppression effect.

**Table 2:** Extraction results of coal dust explosion pressure data.

Test object	Explosion pressure data		Time of occurrence (s)	
	*P*_max_ (MPa)	(d*P*/d*t*)_max_ (MPa/s)	*P*_max_	(d*P*/d*t*)_max_
Coal dust	0.58	32.90	0.625	0.375

### Inhibition effect of mixed explosion suppressants on coal dust explosion pressure

#### Inhibitory effect of mixing CaCO_3_ and KCl on explosion pressure

In previous studies of this paper, the results of coal dust explosion pressure have been obtained. However, there is a noteworthy issue, which is that although the explosion suppression effect of K_2_CO_3_ is better than that of KCl, and the explosion suppression effect of KCl is better than that of CaCO_3_. However, when suppressing industrial dust explosions, cost considerations need to be taken into account. It is unrealistic to blindly use the best explosion suppression agent K_2_CO_3_ without considering cost. Therefore, in the following research, the focus will be on analyzing the coal dust explosion suppression characteristics under different mixed conditions of explosion suppressants. Firstly, in this section of the experiment, two of the three types of explosion suppressants are mixed, and then the three types of explosion suppressants are mixed in the following text. The purpose of the study is to obtain explosion suppression conditions with good explosion suppression effect and low cost by setting up a research plan for mixed explosion suppressants. The average market prices of the procurement costs for the three types of explosion suppressants are shown in Table 3, this price is from China, and if other countries need to refer to it, it can be converted to that country's price.

**Table 3:** Average market purchase prices of three types of explosion suppressants.

Explosion suppressant name	CaCO_3_	KCl	K_2_CO_3_
Active ingredient content	99%	99%	99%
Purchase price (USD/1000 kg)	112	676	1127

It can be seen that the average market purchase price of CaCO_3_ is the lowest, at 112 USD/1000 kg, the average market purchase price of KCl is six times that of CaCO_3_, and K_2_CO_3_ is ten times that of CaCO_3_. It is obvious that although K_2_CO_3_ has the best explosion suppression effect, its average market purchase price is also the highest. Therefore, when using explosion suppressants for coal dust explosion suppression, both purchase cost and explosion suppression effect must be considered simultaneously. This is also the original intention of proposing an experimental plan for mixed explosion suppressants in this article. In the experimental analysis of mixed explosion suppressants in the following text, the focus will also be on comprehensively considering both the explosion suppression effect and purchase cost, and providing reference for obtaining the optimal explosion suppression scheme.

Firstly, mix CaCO_3_ and KCl according to a mass percentage of 50%: 50%, and then mix them with coal dust. The sample mass of coal dust is still 10 g, ensuring a mass concentration of 500 g/m^3^ for coal dust clouds. The experimental results of CaCO_3_ and KCl mixed explosion suppression obtained are shown in Table 4. *m*_1_ represents the mass of two types of explosion suppressants CaCO_3_ and KCl mixed into coal dust. It can be observed that as the mass of the mixed explosion suppressant increases within the range of 0 ~ 5 g, the inhibitory effect on the maximum pressure and maximum pressure rise rate of coal dust explosion continues to increase. After comparing the inhibitory effect of CaCO_3_ and KCl mixed suppressants with that of a single suppressant, it was found that the inhibitory effect of CaCO_3_ and KCl mixed suppressants was between the inhibitory effects of using a single suppressant CaCO_3_ or KCl. The inhibitory effect of mixed explosion suppressants is better than that of single explosion suppressant CaCO_3_, but worse than that of single explosion suppressant KCl. From the experimental data, it can be seen that the maximum explosion pressure and maximum pressure rise rate under the mixed explosion suppression conditions of CaCO_3_ and KCl are always between the data of a single explosion suppressant CaCO_3_ and KCl.

**Table 4:** Coal dust explosion pressure data under mixed explosion suppression conditions of CaCO_3_ and KCl.

Explosion suppressants		*m*_1_ (g)					
		0	1	2	3	4	5
CaCO_3_ and KCl mixed	*P*_max_ (MPa)	0.58	0.51	0.43	0.37	0.33	0.28
	(d*P*/d*t*)_max_ (MPa/s)	32.90	27.21	24.96	21.20	18.66	15.73

**Figure 5:**
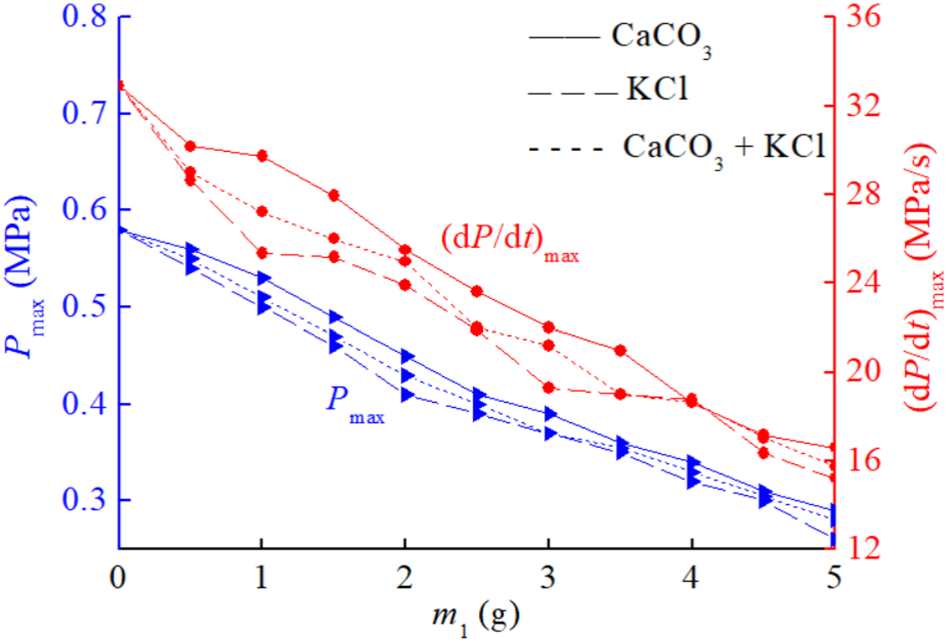
Comparison of inhibitory effects of CaCO_3_ and KCl mixed suppressants and single suppressants.

From the perspective of explosion suppression effect, when CaCO_3_ and KCl are mixed to suppress coal dust explosion, there is no significant synergistic effect between the two explosion suppressants. If there is a significant synergistic effect, the inhibitory effect of mixing two explosion suppressants will be better than using one explosion suppressant alone. The current experimental results indicate that the synergistic effect of mixing CaCO_3_ and KCl is almost non-existent or very inconspicuous. Both types of explosion suppressants have their own independent inhibitory effects, including blocking energy transfer between coal dust particles, reducing the surface temperature of coal dust particles, and so on. The comparison between the explosion suppression curve of CaCO_3_ and KCl mixed conditions and the explosion suppression curve of a single inhibitor is shown in Fig. 5.

Next, an analysis will be conducted from the perspective of combining the explosion suppression effect and the cost of purchasing explosion suppressants. The purchase cost of explosion suppressant CaCO_3_ is relatively low, with a selling price of only 112 USD/1000 kg, but its effectiveness in suppressing coal dust explosion pressure alone is not good. The purchase cost of explosion suppressant KCl is six times higher than that of CaCO_3_, and its suppression effect on coal dust explosion pressure is significantly better than that of CaCO_3_. Both types of explosion suppressants have their own advantages. Analysis suggests that in the case where both types of explosion suppressants are sufficient, in order to ensure safe production, it is recommended to use explosion suppressant KCl as much as possible between explosion suppressants CaCO_3_ and KCl. However, when the explosive suppressant KCl is not sufficient, one method that can be adopted is to mix relatively inexpensive explosive suppressant CaCO_3_ with explosive suppressant KCl and then carry out the explosive suppression operation. Although the suppression effect of mixing CaCO_3_ and KCl is not as good as using KCl alone, it is also much better than using CaCO_3_ alone to suppress explosions. Therefore, the method of suppressing explosions by mixing CaCO_3_ and KCl is worth recommending.

#### Inhibition effect of mixing CaCO_3_ and K_2_CO_3_ on explosion pressure

After obtaining the suppressant effect of CaCO_3_ and KCl mixture on coal dust explosion pressure, in this section, we will consider mixing CaCO_3_ and K_2_CO_3_. The purchase cost of K_2_CO_3_ is ten times that of CaCO_3_, but its inhibitory effect is the best among the three types of explosion suppressants. In order to comprehensively consider the relationship between purchase cost and explosion suppression effect, an experiment was designed to combine CaCO_3_ and K_2_CO_3_ for explosion suppression. This is to find a more suitable explosion suppression scheme and provide theoretical basis for coal mine explosion suppression. CaCO_3_ and K_2_CO_3_ are mixed in a 50%: 50% mass percentage. The mass of coal dust is still 10 g. The experimental data of CaCO_3_ and K_2_CO_3_ mixed explosion suppression are shown in Table 5, m_2_ represents the sum of the masses of CaCO_3_ and K_2_CO_3_ after mixing. It can be found that the explosion suppression effect of CaCO_3_ and K_2_CO_3_ mixed is better than that of CaCO_3_ and KCl mixed, indicating that K_2_CO_3_ plays an important role in the mixed explosion suppression, which is related to the generation of K_2_O, K_2_O_2_, KOH, CO_2_, and H_2_O by K_2_CO_3_ after decomposition. The explosion suppression effect of individual KCl is worse than that of K_2_CO_3_, which inevitably leads to a worse explosion suppression effect of CaCO_3_ and KCl mixed compared to CaCO_3_ and K_2_CO_3_ mixed. K_2_CO_3_ plays a crucial role in mixed explosion suppression.

**Table 5:** Coal dust explosion pressure data under mixed explosion suppression conditions of CaCO_3_ and K_2_CO_3_.

Explosion suppressants		*m*_2_ (g)					
		0	1	2	3	4	5
CaCO_3_ and K_2_CO_3_ mixed	*P*_max_ (MPa)	0.58	0.49	0.40	0.35	0.29	0.23
	(d*P*/d*t*)_max_ (MPa/s)	32.90	25.27	23.81	18.16	16.29	14.83

**Figure 6:**
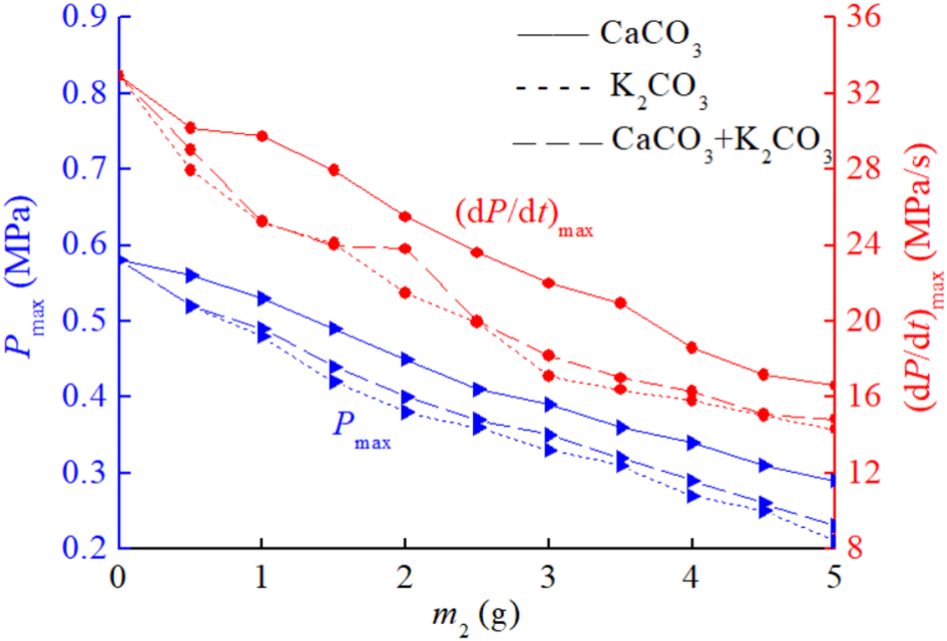
Comparison of inhibitory effects of CaCO_3_ and K_2_CO_3_ mixed suppressants and single suppressants.

Comparing the explosion suppression effect of mixing CaCO_3_ and K_2_CO_3_ with the explosion suppression effect of using K_2_CO_3_ alone, as shown in Fig. 6, it can be seen that the explosion suppression effect of mixing CaCO_3_ and K_2_CO_3_ is not as good as that of using K_2_CO_3_ alone. CaCO_3_ has a much higher melting point than K_2_CO_3_, and when K_2_CO_3_ is thermally decomposed, CaCO_3_ cannot decompose quickly. Therefore, when CaCO_3_ and K_2_CO_3_ are mixed for explosion suppression, CaCO_3_ actually has a certain hindering effect on K_2_CO_3_'s explosion suppression, but CaCO_3_ contributes to the overall mixed explosion suppression effect. Because without adding CaCO_3_, the explosion suppression effect of using only 50% K_2_CO_3_ is not as good as the explosion suppression effect of mixing CaCO_3_ and K_2_CO_3_. For example, in a mixed explosion suppressant of 2 g CaCO_3_ and K_2_CO_3_, containing 1 g K_2_CO_3_, the maximum pressure and maximum pressure rise rate of the mixed explosion suppressant of 2 g CaCO_3_ and K_2_CO_3_ are 0.40 MPa and 23.81 MPa/s, respectively, while the maximum pressure and maximum pressure rise rate of only 1 g K_2_CO_3_ are 0.48 MPa and 25.21 MPa/s, respectively. It is obvious that the mixed explosion suppressant of CaCO_3_ and K_2_CO_3_ has a better effect, and the same is true when the mass of the mixed explosion suppressant is 4 g. Therefore, it can be concluded that when CaCO_3_ and K_2_CO_3_ are mixed for explosion suppression, K_2_CO_3_ plays a key inhibitory role, and CaCO_3_ also plays a certain auxiliary role. Although the mixed explosion suppression effect is not as good as using K_2_CO_3_ alone, it is much better than using CaCO_3_ alone. Therefore, the mixed explosion suppression of CaCO_3_ and K_2_CO_3_ is a worthwhile explosion suppression method to consider.

When CaCO_3_ and K_2_CO_3_ are mixed for explosion suppression, the explosion suppression effect is worse than using K_2_CO_3_ alone, indicating that there is almost no significant synergistic effect or the synergistic effect is very small after the two are mixed. Otherwise, the mixed explosion suppression effect will be better than the effect of using any type of explosion suppressant alone. Finding a hybrid explosion suppression method with good synergistic explosion suppression effect is an important way to optimize hybrid explosion suppression technology. In addition, the explosion suppression effect of CaCO_3_ and K_2_CO_3_ mixed conditions is better than that of using KCl alone. Therefore, from the perspective of purchase cost, the purchase cost of 1000 kg CaCO_3_ and 1000 kg K_2_CO_3_ is lower than that of 2000 kg KCl. Therefore, whether in terms of explosion suppression effect or purchase cost, choosing the method of CaCO_3_ and K_2_CO_3_ mixed explosion suppression is better than using KCl alone for explosion suppression. This is a conclusion that coal mining enterprises can refer to when it comes to safety and explosion prevention.

#### Inhibition effect of mixing KCl and K_2_CO_3_ on explosion pressure

For the three explosion suppressants used in this article, in addition to mixing CaCO_3_ and KCl, CaCO_3_ and K_2_CO_3_, KCl and K_2_CO_3_ can also be mixed to further study the suppression effect on coal dust explosion pressure. KCl and K_2_CO_3_, two types of explosion suppressants, have relatively good explosion suppression effects when used alone. Mix KCl and K_2_CO_3_ in a 50%: 50% mass ratio, and then mix them into coal dust to study the explosion suppression effect. The mass of coal dust is 10 g. The data results of the explosion suppression experiment are shown in Table 6. It can be observed that as the mass of the mixed explosion suppressant KCl and K_2_CO_3_ increases in the range of 0 ~ 5 g, the maximum pressure and maximum pressure rise rate of coal dust explosion decrease continuously. When the mass of the mixed explosion suppressant KCl and K_2_CO_3_ is 5 g, the maximum pressure and maximum pressure rise rate are 0.18 MPa and 12.60 MPa/s, respectively. At this point, the explosion intensity is already very low, and the inhibitory effect of mixed suppressants KCl and K_2_CO_3_ on coal dust explosion is already very obvious, and the fireworks generated by the explosion are also very weak.

**Table 6:** Coal dust explosion pressure data under mixed explosion suppression conditions of KCl and K_2_CO_3_.

Explosion suppressants		*m*_3_ (g)					
		0	1	2	3	4	5
KCl and K_2_CO_3_ mixed	*P*_max_ (MPa)	0.58	0.48	0.39	0.34	0.25	0.18
	(d*P*/d*t*)_max_ (MPa/s)	32.90	25.31	22.47	17.92	14.17	12.60

**Figure 7:**
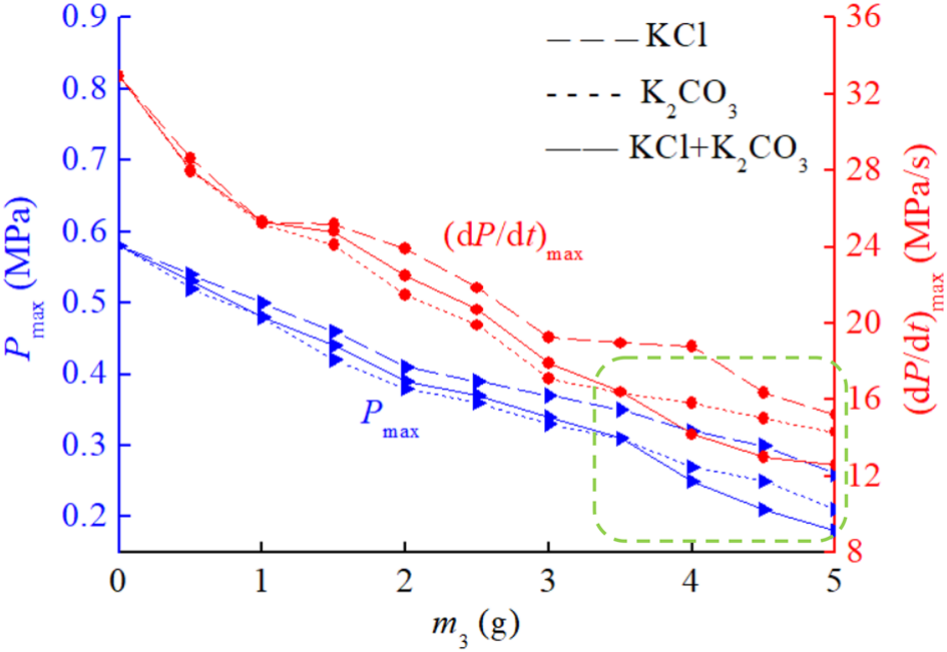
Comparison of inhibitory effects of KCl and K_2_CO_3_ mixed suppressants and single suppressants.

Next, compare the explosion suppression effects of the mixture of KCl and K_2_CO_3_ with those of a single explosion suppressant, as shown in Fig. 7. *m*_3_ represents the sum of the masses of explosion suppressants KCl and K_2_CO_3_. It can be seen that when the sum of the masses of KCl and K_2_CO_3_ is 0 ~ 3.5 g, the inhibitory effect of the mixed explosion suppressant is between the effects of using the two explosion suppressants alone. The inhibitory effect is better than using KCl alone, but slightly worse than using K_2_CO_3_ alone. When the mass of the mixed explosive suppressant is 0 ~ 3.5 g, due to the relatively small amount of explosive suppressant used, the inhibitory effects of KCl and K_2_CO_3_ are independent, and the synergistic effect between the two is very small or almost no synergistic effect. When the mass of KCl and K_2_CO_3_ mixed is 3.5 ~ 5 g, due to the proportion of explosion suppressants mixed into coal dust exceeding 35%, the proportion of explosion suppressants is relatively large, making the inhibitory effect of the mixed explosion suppressants exceed that of using KCl or K_2_CO_3_ alone. At this time, the synergistic effect of the mixed explosion suppressants appears, which is a very desired result in coal dust explosion suppression research and has important theoretical significance for coal dust explosion suppression.

**Figure 8:**
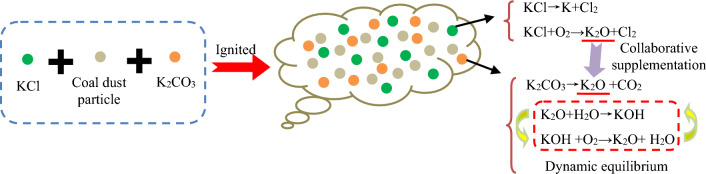
Synergistic suppression process of KCl and K_2_CO_3_ mixed explosion suppressants on coal dust explosion.

The reason for the synergistic effect of mixed explosion suppressants on suppressing coal dust explosion pressure needs to be analyzed. If the amount of explosive suppressant is too small, the synergistic effect will not occur. Secondly, the chemical properties of the explosive suppressant are also important for the synergistic effect. In this experiment, K_2_CO_3_ was selected, which is a very effective explosive suppressant. However, when CaCO_3_ and K_2_CO_3_ were mixed, there was no synergistic effect, while when KCl and K_2_CO_3_ were mixed, there was a synergistic effect. The mixed synergistic explosion suppression process of KCl and K_2_CO_3_ is shown in Fig. 8. This is because KCl has a smaller melting point than CaCO_3_. As a white powder different from K_2_CO_3_, KCl can provide more obstacles to the interaction between K_2_CO_3_ and coal dust in high-temperature environments, making the inhibitory effect of KCl and K_2_CO_3_ mixture particularly prominent. In addition, purchase cost is also a factor to consider. The cost of purchasing 1000 kg of KCl and 1000 kg of K_2_CO_3_ is 1803 USD, while the cost of purchasing 1000 kg of CaCO_3_ and 1000 kg of KCl is 788 USD, the cost of purchasing 1000 kg of CaCO_3_ and 1000 kg of K_2_CO_3_ is 1239 USD, so the cost of purchasing KCl and K_2_CO_3_ is the highest. Although the explosion suppression effect of mixing KCl and K_2_CO_3_ is very good, it is not suitable for coal mining enterprises to use it casually. Considering the purchase cost, the method of using KCl and K_2_CO_3_ mixed explosion suppressants is still limited.

## Conclusions

In this article, the explosion pressure of micrometer scale coal dust is taken as the research object, and the inhibitory effects of three types of explosion suppressants on explosion pressure under mixed use conditions are discussed. The conclusions obtained are as follows.

Based on the pressure curve of coal dust explosion, the explosion process is divided into three stages. 0 ~ 0.375 s is the stage of accelerated release of explosive energy. 0.375 ~ 0.625 s is the stage where the rate of increase in explosion pressure decreases. After 0.625 s, it is the stage of explosive energy dissipation. After the explosion, the volatile decreased the most, and the ash increased by 238.92%. The mass concentration of coal dust cloud with the highest explosion pressure is 500 g/m^3^, and excessive or insufficient concentration is not conducive to the release of explosion energy.

The study on the suppression of coal dust explosion pressure by mixed explosion suppressants shows that the suppression effect of CaCO_3_ and KCl mixed is between the effects of using the two alone. The explosion suppression effect of mixing CaCO_3_ and K_2_CO_3_ is better than that of mixing CaCO_3_ and KCl, and is worse than the explosion suppression effect of using K_2_CO_3_ alone, indicating that K_2_CO_3_ plays a key role in the mixed explosion suppression.

It is found that the synergistic effect of KCl and K_2_CO_3_ mixed explosion suppression is due to the fact that during the explosion suppression process of K_2_CO_3_, KCl can generate K_2_O, which plays an auxiliary inhibitory role. Considering procurement costs, hybrid explosion suppression is a method worth paying attention to and adopting.

## Data Availability

All data generated during this study are included in this published article.
